# Serotonin Availability Shapes the Effects of Phenelzine on Inflammatory Response and Gene Expression in Macrophages

**DOI:** 10.7759/cureus.70912

**Published:** 2024-10-05

**Authors:** Saeed Mahdianipur, Mahafarin Maralani, Saeed Mohammadi, Seyed Reza Khandoozi, Afifeh Jaefari, Homa Davoodi

**Affiliations:** 1 Immunology, Golestan University of Medical Sciences, Gorgan, IRN; 2 Oncology, Atlantic Cancer Research Institute, Dr. Georges-L.Dumont University Hospital Centre, Moncton, CAN; 3 Immunology, Natural and Medical Sciences Research Center, University of Nizwa, Nizwa, OMN; 4 Radiation Oncology, Cancer Research Center, Golestan University of Medical Sciences, Gorgan, IRN; 5 Medical Biotechnology, Cancer Research Center, Golestan University of Medical Sciences, Gorgan, IRN; 6 Medical Immunology, Cancer Research Center, Golestan University of Medical Sciences, Gorgan, IRN

**Keywords:** ahr, inflammatory response, monoamine oxidase, phenelzine, serotonin

## Abstract

Background

Serotonin (5-HT) is a neurotransmitter with extensive physiological influence in the central nervous system (CNS) and various behavioral and biological functions, including immune regulation through 5-HT receptors (5-HTRs) expressed by immune cells. A variety of serotonin-modulating drugs have been developed to treat neurological disorders. Phenelzine, a drug indicated for the management of treatment-resistant depression, is a potent, non-selective inhibitor of monoamine oxidase (MAO), the enzyme that metabolizes serotonin to 5-hydroxyindole acetic acid (5-HIAA). Given the emerging evidence of a bidirectional link between depression and inflammation, as well as the potential therapeutic applications of serotonin-modulating drugs in autoimmune diseases and cancer, our study investigated the pro-inflammatory and anti-inflammatory factors influenced by serotonin and phenelzine.

Methodology

We conducted experiments on RAW264.7 macrophages, exposing them to various combinations and concentrations of serotonin, 5-HIAA, and phenelzine. We assessed the relative gene expression of monoamine oxidase-A (MAO-A), CYP1B1, cyclooxygenase-2 (COX-2), and inducible nitric oxide synthase (iNOS) using real-time PCR and measured the production of IL-6, TNF-α, and IL-10 cytokines using enzyme-linked immunosorbent assays (ELISA).

Results

Our findings revealed that phenelzine can downregulate genes associated with the production of reactive oxygen and nitrogen species, reduce aryl hydrocarbon receptor (AHR)-related gene expression induced by serotonin, and enhance the production of inflammatory cytokines. These effects were significantly influenced by the concentration of available serotonin.

Conclusions

Our study demonstrates that various mechanisms, including AHR activation, modulation of reactive oxygen and nitrogen species production, and others, in addition to the increased availability of serotonin due to phenelzine treatment, can significantly influence the inflammatory state.

## Introduction

Serotonin (5-HT) is well-known as a neurotransmitter, but the majority of the body’s serotonin is produced by enterochromaffin cells in the gastrointestinal tract. Immune cells can interact with serotonin released from these enterochromaffin cells in the gastrointestinal mucosa or from platelets, participating in immune regulation through various 5-HT receptors (5-HTRs) that influence both innate and adaptive immune systems [1,2]. The influence of serotonin on immune cells is significantly dependent on factors like the cell type, the specific subtype of 5-HTR involved, and the disease context. Consequently, it is difficult to definitively categorize serotonin's role as either pro-inflammatory or anti-inflammatory [2].

One of the few consistent findings in patients with more severe forms of depression is lower plasma levels of tryptophan, the amino acid precursor to serotonin. Inflammation can induce depression in susceptible individuals by lowering plasma tryptophan levels and subsequently diminishing brain serotonin activity [3,4]. Indeed, data suggests that depression and anxiety often co-occur with inflammatory bowel disease (IBD), and the connection between these mental health conditions and inflammatory disease appears to be bidirectional. This means that IBD can contribute to the onset of depression and anxiety, while these mental health issues can also affect the progression of IBD [5].

Serotonin-modulating drugs have primarily been designed to treat neurological diseases; however, several studies have indicated their potential application in autoimmune diseases and cancer [2,6,7], Among these drugs, monoamine oxidase inhibitors (MAOIs), first introduced in the 1950s, have shown effectiveness in treating various forms of depression. MAOIs are also prescribed for other neurological disorders, including panic disorder, social phobia, and atypical depression [8].

Phenelzine, an FDA-approved medication primarily prescribed for depression, is a potent non-selective MAOI, Interestingly, it has also shown effectiveness in patients with biochemical recurrent castrate-sensitive prostate cancer [6]. Furthermore, in a mouse model of autoimmune encephalomyelitis (EAE), daily treatment with phenelzine, starting seven days after disease induction, resulted in delayed onset of EAE, reduced disease severity during its chronic phase, and was associated with significant improvements in exploratory behavior. Additionally, it showed promise as a potential treatment for symptoms resembling sickness and depression [7].

Monoamine oxidase (MAO) enzymes degrade monoamines, producing hydrogen peroxide (H₂O₂) as a byproduct. This process elevates reactive oxygen species (ROS) levels in tumor-associated macrophages (TAMs), leading to their immunosuppressive polarization [9]. Inhibiting MAO enzymes has been shown to reduce the expression of cyclooxygenase-2 (COX-2) and inducible nitric oxide synthase (iNOS) in autoimmune disease contexts [10,11].

The aryl hydrocarbon receptor (AHR), a ligand-activated transcription factor, can be triggered by various small molecules from sources such as diet, microorganisms, metabolism, and pollutants. AHR signaling serves as a crucial link between environmental and metabolic factors and immune responses, as it is widely expressed in different immune cells [12]. While it has been suggested that serotonin activates the AHR [13], there is a lack of empirical evidence regarding the effect of phenelzine on AHR activation. However, we know that phenelzine, as a monoamine oxidase (MAO) inhibitor, can inhibit the production of the serotonin-derived metabolite 5-hydroxyindole acetic acid (5-HIAA), which activates AHR [14]. Additionally, AHR signaling has been linked to the enhancement of IL-10 expression in human macrophages and LPS-induced RAW264.7 cells, highlighting its role in immune regulation [15,16].

Macrophage cells express various serotonin receptors, including 1A, 1B, 1E, 2A, 2B, 2C, 3, 4, and 7, along with enzymes like tryptophan hydroxylase (TPH) and the serotonin transporter (SERT). Numerous studies have confirmed the expression of serotonin receptors, particularly 1B and 2B, as well as the TPH enzyme and the SERT in macrophage-like cell lines such as RAW264.7. This indicates the presence of a functional serotonin system within macrophages, suggesting its potential role in immune responses and other cellular functions [17-19]. However, the effects of serotonin on immune cells are context-dependent, varying with specific conditions and signaling contexts [20].

Given the bidirectional relationship between inflammation-related diseases and depression and the potential effects of antidepressant drugs on inflammatory conditions and macrophage polarization through modulation of the serotonergic system and other mechanisms, this study aimed to investigate the influence of phenelzine at varying serotonin concentrations on IL-6, TNF-α, and IL-10 production as key pro-inflammatory and anti-inflammatory cytokines released from macrophages. Additionally, we examined genes associated with ROS and reactive nitrogen species (RNS), including COX-2, iNOS, and CYP1A1 as an indicator of AHR activation, and monoamine oxidase-A (MAO-A) in RAW264.7 macrophage cells.

## Materials and methods

Cell culture and treatment

RAW264.7 cells (Pasteur Institute, Iran) were cultured in a humidified CO_2_ incubator (5% vol.) at 37°C in Roswell Park Memorial Institute Medium (RPMI), from BioIdea, Iran, supplemented with 5% (v/v) fetal bovine serum (FBS), from BioIdea, Iran and appropriate antibiotics (100 IU/mL penicillin and 100 µg/mL streptomycin), from BioIdea, Iran. We assessed the cytotoxicity of all treatments at different concentrations selected based on those used in similar prior studies, using the 3-(4,5-dimethylthiazol-2-yl)-2,5-diphenyltetrazolium bromide (MTT) assay. The treatment group concentrations were selected based on MTT assay results, which indicated no cytotoxicity at these levels (data not shown).

The RAW264.7 cells were divided into different groups, with a concentration of 10^6^ cells per 1 mL of culture medium, including two control groups treated with corresponding solvents: 0.03% dimethyl sulfoxide (DMSO) for the 5-HIAA-treated groups and 0.85% water for the serotonin- and phenelzine-treated groups. The treatment groups included two different concentrations of serotonin (0.1 µM and 10 µM), 10 µM 5-HIAA, 1 µM phenelzine (SIGMA Chemical Company, St. Louis, MO, USA), and combinations of phenelzine with the two different serotonin concentrations and 10 µM 5-HIAA. All groups were treated for 24 hours. Additionally, before the main treatments, all groups were pre-treated with 100 ng/mL LPS for 24 hours. A total of three biological replicates were employed to ensure statistical validity.

RNA extraction and real-time quantitative PCR (RT-qPCR)

RNA extraction was carried out using a chloroform-based method with TRIzol lysis buffer (Yekta Tajhiz Azma, Iran), following the manufacturer's instructions. A total of 10^6^ cells were used for the extraction. The quality and concentration of the total RNA were assessed using the Nanodrop 2000 spectrophotometer (Denovix, DS-11, USA). The total RNA was then standardized to a concentration of 1000 ng/μL and stored at -80°C for future use.

After DNase I treatment (Yekta Tajhiz Azma, Iran), 1 µg of the standardized RNA sample was utilized for complementary DNA (cDNA) synthesis using the cDNA Synthesis Kit (Yekta Tajhiz Azma, Iran), following the manufacturer's guidelines. Messenger RNA quantification for genes including CYP1A1, as an indicator of AHR activation, MAO-A, COX-2, and iNOS was conducted using the Applied Biosystems StepOne Plus Real-Time PCR System (ThermoFisher Scientific, USA), followed by melting curve analyses. The gene expression analysis employed the Sina Green HS-qPCR Mix (Sinaclon, Iran) as per the manufacturer's instructions and primers specifically designed for the mentioned genes (Gene Fanavaran, Tehran, Iran), considering spanning exon junctions. The primer sequences for these genes, along with their respective annealing temperatures, are provided in Table 1. Glyceraldehyde 3-phosphate dehydrogenase (GAPDH) served as a housekeeping gene and was concurrently quantified to normalize the results. Relative mRNA expression fold changes were calculated and plotted using the 2^-ΔΔCT^ formula.

**Table 1:** Primer sequences and annealing temperatures for RT-qPCR iNOS: Nitric oxide synthase; COX-2: Cyclooxygenase-2; MAO-A: Monoamine oxidase-A; GAPDH: Glyceraldehyde 3-phosphate dehydrogenase.

Gene	Forward (5′–3′)	Reverse (5′–3′)	Annealing temperature (°C)
iNOS	ACTACTGCTGGTGGTGACAA	CCTGAAGGTGTGGTTGAGTTC	54
COX-2	ATCAGGTCATTGGTGGAGAGG	ATGGTGGCATACATCATCAGAC	57
MAO-A	ATGCTGAGGAATGGGACAAG	GGTGACTGAGAATATCCGAGAG	53
CYP1A1	CATCACAGACAGCCTCATTGAGC	CTCCACGAGATAGCAGTTGTGAC	60
GAPDH	GGTTGTCTCCTGCGACTTCA	CCTGTTGCTGTAGCCGTATTC	57

Measurement of cytokine levels

The commercially available enzyme-linked immunosorbent assay (ELISA) kit (Karmania Pars Gene Company, Kerman, Iran) was used to determine the supernatant level of IL-6, TNF-α, and IL-10 in all groups following the manufacturer's protocol. The optical density of each sample was measured at the wavelength of 450 nm using BioTek ELISA reader ELX800 (BioTek, VT, USA). The results were reported as picograms per milliliter (pg/mL).

Statistical analysis

The results, derived from three replicates, are presented as the median and interquartile range (IQR). Data organization and visualization were performed using Microsoft Excel software (Microsoft Corp., Redmond, WA). Statistical analyses were conducted using SPSS software (IBM Corp., Armonk, NY). To ensure comparability, the results were normalized to the control cells treated with the corresponding solvent. The Shapiro-Wilk test was utilized to assess normality. Statistical analysis was carried out using the independent sample Kruskal-Wallis test due to the non-normal distribution of data for overall group comparisons. Post-hoc pairwise comparisons were then performed using Dunn's test to identify specific differences between groups. A p-value threshold of less than 0.05 was considered statistically significant, ensuring a robust evaluation of both global and pairwise differences among the experimental groups.

## Results

Modulation of MAO-A expression by phenelzine and serotonin

**Figure 1:**
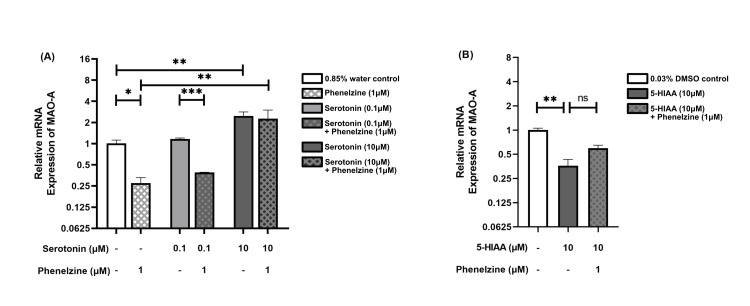
Relative mRNA expression of MAO-A in LPS-stimulated RAW264.7 cells The bar chart presents the median and interquartile range (IQR) of relative mRNA expression levels of MAO-A across different treatment groups. Each column represents a distinct treatment group, with the median values indicated by the height of the bars and the IQR depicted by the error bars. Below each column, two rows detail the specific concentrations administered to each group. The dash (“-”) denotes that the respective treatment group did not receive the specified treatment. (A) Relative mRNA expression of MAO-A in groups treated with phenelzine, two concentrations of serotonin, and their combination, compared to a 0.85% water control. (B) Relative mRNA expression of MAO-A in groups treated with 5-HIAA and its combination with phenelzine, compared to a 0.03% DMSO control. *p < 0.05; **p < 0.01; ***p < 0.001. MAO-A: Monoamine oxidase-A; LPS: Lipopolysaccharide; DMSO: Dimethyl sulfoxide; 5-HIAA: 5-hydroxyindole acetic acid; ns: Not significant.

Treatment of LPS-stimulated RAW264.7 cells with phenelzine (1 μM) led to a significant decrease in MAO-A expression, approximately threefold lower compared to the control group (p = 0.018). Similarly, when comparing groups treated with 0.1 μM of serotonin, MAO-A expression decreased approximately threefold (p = 0.005). Notably, serotonin at 10 μM increased MAO-A expression approximately twofold compared to the control group (p = 0.005) and showed a remarkable eightfold increase in phenelzine (1 μM)-treated groups (p = 0.005) (Figure 1, Panel A).

In contrast, treating RAW264.7 cells with 5-HIAA (10 μM) resulted in an approximate threefold decrease in MAO-A expression compared to the control group (p = 0.005). Interestingly, the addition of phenelzine in 5-HIAA (10 μM)-treated groups did not induce a significant change in MAO-A expression (Figure 1, Panel B).

Modulation of CYP1A1 expression by phenelzine and serotonin

**Figure 2:**
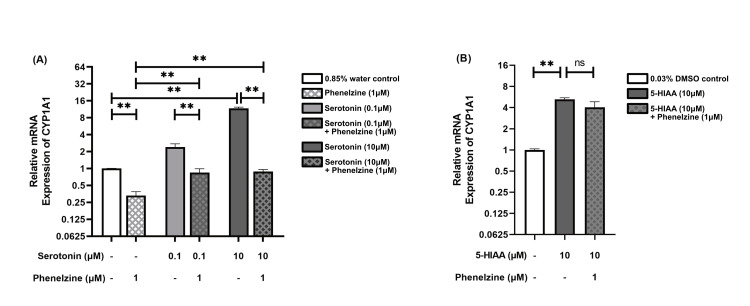
Relative mRNA expression of CYP1A1 in LPS-stimulated RAW264.7 cells The bar chart presents the median and interquartile range (IQR) of relative mRNA expression levels of CYP1A1 across different treatment groups. Each column represents a distinct treatment group, with the median values indicated by the height of the bars and the IQR depicted by the error bars. Below each column, two rows detail the specific concentrations administered to each group. The dash (“-”) denotes that the respective treatment group did not receive the specified treatment. (A) Relative mRNA expression of CYP1A1 in groups treated with phenelzine, two concentrations of serotonin, and their combination, compared to a 0.85% water control. (B) Relative mRNA expression of CYP1A1 in groups treated with 5-HIAA and its combination with phenelzine, compared to a 0.03% DMSO control. *p < 0.05; **p < 0.01; ***p < 0.001. LPS: Lipopolysaccharide; DMSO: Dimethyl sulfoxide; 5-HIAA: 5-Hydroxyindole acetic acid; ns: Not significant.

Treating LPS-stimulated RAW264.7 cells with phenelzine (1 μM) resulted in approximately a threefold decrease in CYP1A1 expression (p = 0.003) compared to the control group. Similarly, in the serotonin-treated groups, CYP1A1 expression decreased by approximately threefold (p = 0.005) at 0.1 μM and by approximately 13-fold (p = 0.005) at 10 μM. Notably, serotonin (10 μM) significantly increased CYP1A1 expression compared to the control group, approximately 12-fold (p = 0.005). Additionally, serotonin increased CYP1A1 expression in phenelzine (1 μM)-treated groups at both low (0.1 μM) and high (10 μM) concentrations, approximately twofold (p = 0.009), indicating that the ability of serotonin to increase CYP1A1 expression in proportion to concentration is reduced by phenelzine treatment (Figure 2, Panel A).

Treatment with 5-HIAA (10 μM) led to an overexpression of CYP1A1 compared to the control group, approximately fivefold (p = 0.005). However, no significant change was observed in phenelzine-treated groups when co-treated with 5-HIAA (10 μM), unlike the pattern seen in the serotonin (10 μM)-treated groups (Figure 2, Panel B).

Phenelzine's impact on COX-2 expression in serotonin-treated RAW264.7 cells

**Figure 3:**
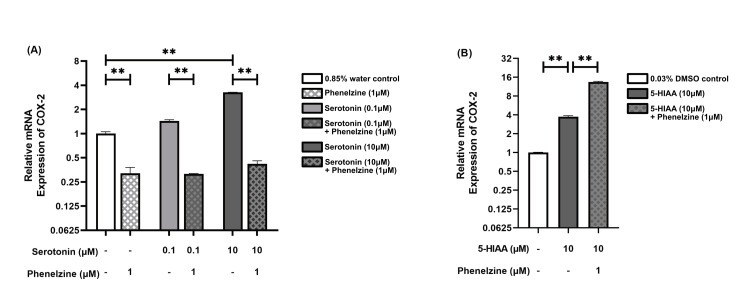
Relative mRNA expression of COX-2 in LPS-stimulated RAW264.7 cells The bar chart presents the median and interquartile range (IQR) of relative mRNA expression levels of COX-2 across different treatment groups. Each column represents a distinct treatment group, with the median values indicated by the height of the bars and the IQR depicted by the error bars. Below each column, two rows detail the specific concentrations administered to each group. The dash (“-”) denotes that the respective treatment group did not receive the specified treatment. (A) Relative mRNA expression of COX-2 in groups treated with phenelzine, two concentrations of serotonin, and their combination, compared to a 0.85% water control. (B) Relative mRNA expression of COX-2 in groups treated with 5-HIAA and its combination with phenelzine, compared to a 0.03% DMSO control. *p < 0.05; **p < 0.01; ***p < 0.001. LPS: Lipopolysaccharide; DMSO: Dimethyl sulfoxide; 5-HIAA: 5-Hydroxyindole acetic acid.

When LPS-stimulated RAW264.7 cells were treated with phenelzine (1 μM), there was an approximately threefold decrease in COX-2 expression compared to the control group (p = 0.005). Furthermore, phenelzine (1 μM) decreased COX-2 expression approximately four times (p = 0.001) in groups treated with 0.1 μM serotonin. In groups treated with 10 μM serotonin, phenelzine (1 μM) led to an approximately eightfold reduction in COX-2 relative expression (p = 0.005). Interestingly, COX-2 expression remained relatively consistent in phenelzine-treated groups, irrespective of the serotonin concentration. At a 10 μM serotonin concentration, there was a significant approximately threefold increase in COX-2 relative expression compared to the control group (p = 0.005). However, 0.1 μM serotonin did not yield any significant difference in COX-2 expression (Figure 3, Panel A).

Treatment with 5-HIAA (10 μM) resulted in an approximately fourfold overexpression of COX-2 compared to the control group (p = 0.002). Additionally, a significant increase of approximately three times in COX-2 expression was observed when phenelzine was administered to -HIAA (10 μM)-treated groups (p = 0.002), as shown in Figure 3 (Panel B).

Serotonin's influence on phenelzine-mediated iNOS regulation in RAW264.7 cells

**Figure 4:**
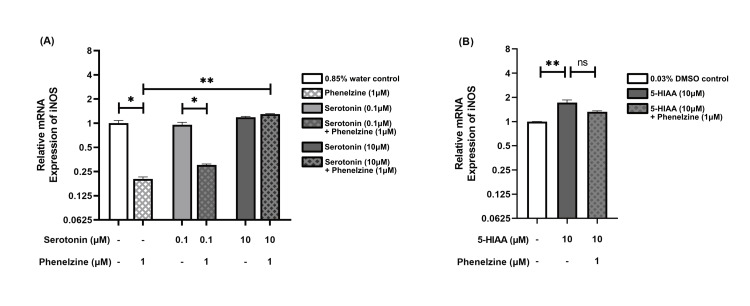
Relative mRNA expression of iNOS in LPS-stimulated RAW264.7 cells The bar chart presents the median and interquartile range (IQR) of relative mRNA expression levels of iNOS across different treatment groups. Each column represents a distinct treatment group, with the median values indicated by the height of the bars and the IQR depicted by the error bars. Below each column, two rows detail the specific concentrations administered to each group. The dash (“-”) denotes that the respective treatment group did not receive the specified treatment. (A) Relative mRNA expression of iNOS in groups treated with phenelzine, two concentrations of serotonin, and their combination, compared to a 0.85% water control. (B) Relative mRNA expression of iNOS in groups treated with 5-HIAA and its combination with phenelzine, compared to a 0.03% DMSO control. *p < 0.05; **p < 0.01; ***p < 0.001. LPS: Lipopolysaccharide; DMSO: Dimethyl sulfoxide; 5-HIAA: 5-Hydroxyindole acetic acid; ns: Not significant; iNOS: Inducible nitric oxide synthase.

Treatment of LPS-stimulated RAW264.7 cells with phenelzine (1 μM) resulted in an approximate fivefold decrease in iNOS expression compared to the control group (p = 0.018). When comparing groups treated with serotonin at a 0.1 μM concentration, this reduction was approximately threefold (p = 0.041). Notably, no change was observed in groups treated with serotonin at a 10 μM concentration when co-administered with phenelzine. This suggests that serotonin may diminish the impact of phenelzine on reducing iNOS expression, and serotonin appears to neutralize this effect at the highest concentration (10 μM). However, serotonin at both concentrations (0.1 and 10 μM) did not significantly alter iNOS relative expression compared to the control group. Yet, when comparing groups treated with phenelzine, serotonin at a concentration of 10 μM induced a significant (p < 0.005) approximately sixfold overexpression of iNOS (Figure 4, Panel A).

In contrast to serotonin, treatment with 5-HIAA (10 μM) led to an approximate twofold overexpression of iNOS compared to the control group (p = 0.005). Interestingly, no significant change was observed when phenelzine was administered to 5-HIAA (10 μM)-treated groups (Figure 4, Panel B).

Cytokine production modulation in RAW264.7 cells by phenelzine, serotonin, and 5-HIAA

**Figure 5:**
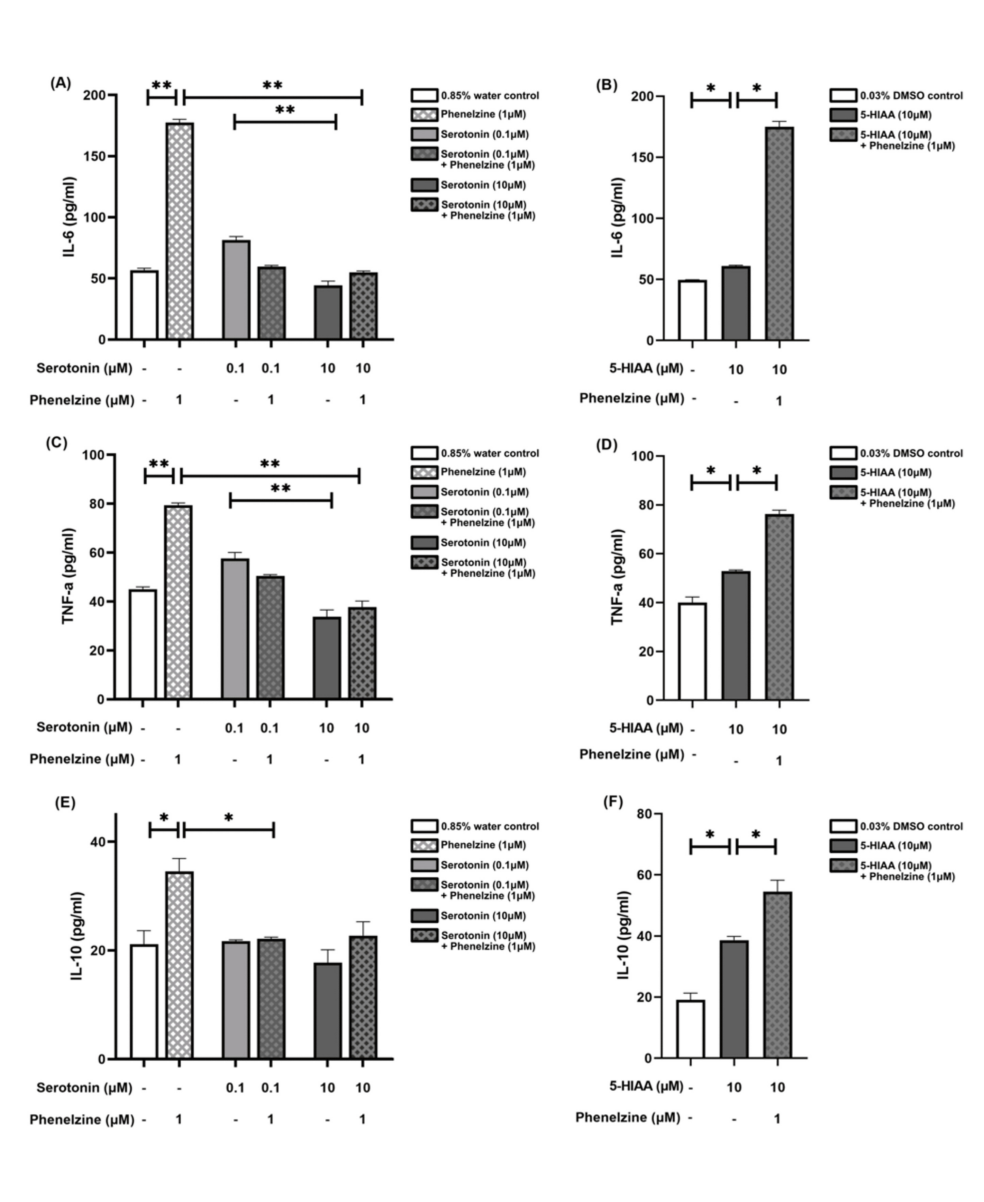
Cytokine production in LPS-stimulated RAW264.7 cells measured by ELISA The bar chart presents the median and interquartile range (IQR) of cytokine production across different treatment groups in picograms per milliliter. Each column represents a distinct treatment group, with the median values indicated by the height of the bars and the IQR depicted by the error bars. Below each column, two rows detail the specific concentrations administered to each group. The dash (“-”) denotes that the respective treatment group did not receive the specified treatment. (A, C, and E) Production of IL-6, TNF-α, and IL-10 in groups treated with phenelzine, two concentrations of serotonin, and their combination, compared to a 0.85% water control. (B, D, and F) Production of IL-6, TNF-α, and IL-10 in groups treated with 5-HIAA and its combination with phenelzine, compared to a 0.03% DMSO control. *p < 0.05; **p < 0.01; ***p < 0.001. LPS: Lipopolysaccharide; DMSO: Dimethyl sulfoxide; 5-HIAA: 5-Hydroxyindole acetic acid; ELISA: Enzyme-linked immunosorbent assays.

Treating LPS-stimulated RAW264.7 cells with phenelzine (1 μM) increased IL-6, TNF-α, and IL-10 production by approximately three (p = 0.004), twofold (p = 0.004), and 1.5-fold (p = 0.016), respectively, compared to the control group. However, serotonin in phenelzine (1 μM)-treated groups decreased IL-6 and TNF-α production at a 10 μM concentration and reduced IL-10 production at a 0.1 μM concentration, approximately returning to control levels (p = 0.008, p = 0.008, and p = 0.029, respectively), as shown in Figure 5 (Panels A, C, and E).

An increase in the production of all three cytokines was observed with 5-HIAA treatment (10 μM) (p = 0.03), which was more apparent in IL-10 production compared to the other two cytokines. Similarly, an increase in the production of all three cytokines was noted with phenelzine treatment in 5-HIAA-treated groups (p = 0.03), which was more apparent in IL-6 production compared to the other two cytokines (Figure 5, Panels B, D, and F). A higher concentration of serotonin (10 μM) decreased IL-6 and TNF-α production compared to a lower concentration (0.1 μM) by approximately two times (p = 0.004), as shown in Figure 5 (Panels A and C).

## Discussion

This study aimed to investigate the effect of phenelzine, an antidepressant drug, on the inflammatory state of RAW264.7 macrophages under varying levels of serotonin. The results of our study indicated that phenelzine increased the production of inflammatory cytokines in the absence of serotonin. However, in the presence of moderate and high concentrations of serotonin, phenelzine did not significantly alter inflammatory cytokines but reduced genes associated with reactive oxygen and nitrogen species.

Our data demonstrated that treating RAW264.7 macrophages with phenelzine (1μM) resulted in a decrease in MAO-A gene expression compared to the control group. This effect was also observed in groups treated with both phenelzine and serotonin at a concentration of 0.1 μM. However, the exact mechanism by which phenelzine reduces MAO-A expression remains unclear. Interestingly, we observed similarities in the patterns of gene expression changes for iNOS, COX-2, and MAO-A. Treating RAW264.7 cells with phenelzine (1μM) led to a decrease in the gene expression of iNOS and COX-2 compared to the control group. This effect was also evident in groups treated with both phenelzine and serotonin at a concentration of 0.1 μM. However, in groups treated with 10 μM of serotonin, phenelzine only decreased COX-2 expression.

Phenelzine is an inhibitor of the MAO enzyme, which is involved in ROS production mainly by generating H_2_O_2_. A study investigating the impact of the MAO enzyme and its inhibitors on the regulation of neurotransmitters associated with chronic inflammation and joint and systemic organ damage in rheumatoid arthritis (RA) found that inhibiting the MAO enzyme effectively reduces joint inflammation by inhibiting COX-2 [10]. Additionally, MAO inhibition has been shown to decrease the expression of key inflammatory markers, including IL-1β, IL-6, TNF-α, iNOS, and COX-2 at both mRNA and protein levels in primary microglia [11]. This inhibition also mitigates inflammation in murine models by blocking the H_2_O_2_-mediated NF-κB-COX-2 signaling pathway, thereby reducing leukocyte extravasation and COX-2 levels [21].

We assessed the expression of the CYP1A1 gene using a real-time PCR technique as an indicator of AHR receptor activation levels. Similar to our observation with MAO-A, we noted a resemblance in the patterns of gene expression changes for iNOS, COX-2, and CYP1A1. In the case of CYP1A1 expression, our results indicate that phenelzine (1 μM) led to a decrease in CYP1A1 expression compared to the control group. This reduction in CYP1A1 expression was also evident in groups treated with serotonin at concentrations of 0.1 and 10 μM. Lower CYP1A1 expression suggests reduced AHR activation, which may result in decreased levels of ROS and RNS.

A study has reported that 2,3,7,8-tetrachlorodibenzo-p-dioxin (TCDD), a potent AHR activator, increased intracellular calcium concentration, initiating a series of reactions that ultimately led to the activation of COX-2 and the accumulation of inflammatory mediators. The production of ROS by cytochromes P450 is linked to a catalytic cycle of enzymes, specifically involving a phenomenon known as "uncoupling” [22]. Additionally, an AHR-dependent increase in iNOS expression is reported in airway epithelial cells and macrophages [23].

Our data revealed a significant increase in TNF-α and IL-6 levels in phenelzine-treated RAW264.7 cells, despite the decrease in COX-2 and iNOS gene expression. This suggests that the observed increase may be mediated by mechanisms other than the reduction of ROS and RNS. One potential mechanism could be the inhibition of lysine-specific histone demethylase 1A (LSD1), which can regulate macrophage polarization toward a more inflammatory M1 phenotype [24]. It is important to note that there is also evidence suggesting an anti-inflammatory role for phenelzine. Phenelzine has been shown to exert anti-inflammatory effects by reducing reactive oxygen species and pro-inflammatory cytokines in LPS-treated rats [25]. Additionally, phenelzine’s ability to increase gamma-aminobutyric acid (GABA) and biogenic amines, which possess anti-inflammatory properties, contributes to its clinical benefits in EAE disease [7].

It was observed that IL-10 production increased in phenelzine-treated RAW264.7 cells, which parallels the changes seen in TNF-α and IL-6 levels. While IL-10 is typically recognized for its role in reducing inflammation and immune responses, it is crucial to acknowledge that its effects can vary significantly depending on the specific context. In certain cases, it can have pro-inflammatory effects. For instance, Lauw et al. found that IL-10 can increase inflammation in individuals exposed to endotoxins by heightening IFNγ production [26]. Similarly, high-dose IL-10 in Crohn’s disease patients increased IFNγ production [27].

An increase in MAO-A gene expression was noted when RAW264.7 cells were treated with serotonin at a concentration of 10 μM, compared to the control group and groups treated with 1 μM phenelzine. Additionally, treating RAW264.7 cells with serotonin at a 10 μM concentration led to an increase in COX-2 expression compared to the control group. In phenelzine-treated groups, serotonin increased the expression of the iNOS gene at a 10 μM concentration. This increase in COX-2 expression induced by serotonin may be linked to its breakdown by MAO-A, leading to the generation of H_2_O_2_ as a byproduct [9].

An increase in AHR activity was indicated by the elevated expression of CYP1A1 when cells were exposed to serotonin at a concentration of 10 μM compared to the control group. Additionally, there was an increased relative expression of CYP1A1 in cells treated with serotonin at both 10 and 0.1 μM concentrations when compared to phenelzine-treated groups. These findings suggest that serotonin can influence AHR activation and CYP1A1 expression in a dose-dependent manner, and its interactions with phenelzine may further modulate these effects.

The MAO-A enzyme breaks down serotonin and generates 5-HIAA, which can activate AHR [10]. Our data revealed that similar to serotonin, treating RAW264.7 cells with 5-HIAA (10 μM) also increased CYP1A1 expression. However, in contrast, when cells were co-treated with 5-HIAA and phenelzine (an inhibitor of the MAO enzyme), there was no significant change in CYP1A1 expression. Conversely, in groups where serotonin was co-administered with phenelzine, phenelzine decreased CYP1A1 expression. This suggests that serotonin's ability to activate AHR may be mediated by the MAO enzyme's conversion of serotonin into 5-HIAA, Notably, 10 μM serotonin increased CYP1A1 expression by eight times compared to the control group, while 5-HIAA increased it by four times.

Additionally, serotonin increased CYP1A1 expression in phenelzine-treated groups. Therefore, we cannot conclusively state that serotonin's ability to activate AHR is solely limited to its conversion into 5-HIAA. Serotonin may participate in AHR activation through both direct and indirect mechanisms, as several suggested mechanisms exist for serotonin-mediated AHR activation.

Similar to serotonin, we observed an increase in iNOS and COX-2 expression when treating RAW264.7 cells with 5-HIAA (10 μM) compared to the control group. This increase is possibly mediated by heightened AHR activity induced by 5-HIAA. In groups that were also treated with 5-HIAA, phenelzine did not change iNOS expression, whereas it reduced iNOS expression in groups co-treated with 0.1 μM of serotonin. This suggests that phenelzine, by inhibiting 5-HIAA production in serotonin-treated groups, prevents AHR activation and subsequently suppresses iNOS expression.

However, in 5-HIAA-treated groups, phenelzine cannot prevent AHR activation, as 5-HIAA molecules are already present in the cell culture medium. Interestingly, phenelzine increased COX-2 expression in 5-HIAA-treated groups. While this observation is intriguing, further studies are required to provide a convincing explanation for this unexpected result.

Our data revealed that treating RAW264.7 cells with serotonin increased MAO-A expression compared to the control groups. Conversely, 5-HIAA decreased MAO-A expression, and this effect occurred regardless of whether the cells were also treated with phenelzine. This effect is likely mediated by a positive feedback loop, in which increased MAO-A expression responds to the heightened availability of its substrate, serotonin, while decreased MAO-A expression corresponds to the increased availability of its product, 5-HIAA.

We noticed that treating RAW264.7 cells with serotonin at both concentrations did not result in any significant changes in IL-6, TNF-α, and IL-10 production compared to the control group. However, there was a decrease in IL-6 and TNF-α production when the concentration of serotonin was increased to 10 μM compared to 0.1 μM. Furthermore, in groups that were also treated with phenelzine, serotonin at both concentrations (10 and 0.1 μM) reduced the production of IL-6, TNF-α, and IL-10, bringing them down to control levels in LPS-induced RAW264.7 cells. These findings suggest that serotonin, particularly in the presence of phenelzine, has a regulatory effect on the production of these cytokines in an LPS-induced inflammatory context.

An increase in the production of IL-6, TNF-α, and IL-10 by 5-HIAA was noted, with a more pronounced effect on IL-10 production compared to the other two cytokines. As mentioned previously, 5-HIAA activates AHR, which can enhance IL-10 expression in human macrophages and LPS-induced RAW264.7 macrophages [15,16]. Indeed, the effects of AHR activation on the production of inflammatory cytokines can vary across studies. However, it is noteworthy that a particular study reported that AHR agonists, including 2,3,7,8-tetrachlorodibenzo-p-dioxin (TCDD), IP, and 6-formylindolo[3,2-b]carbazole (FICZ), significantly induced the expression of TNF-α in RAW264.7 cells. This highlights the complexity of AHR signaling and its potential to influence the production of inflammatory cytokines in a context-dependent manner [28].

In the 5-HIAA-treated groups, phenelzine resulted in increased production levels of IL-6, TNF-α, and IL-10, with IL-6 showing a more pronounced effect compared to the other two cytokines. As previously discussed, this could potentially be attributed to phenelzine's inhibition of LSD1. Similar to phenelzine, serotonin's impact on the expression of genes related to ROS and RNS production exhibited an opposite trend compared to IL-6 and TNF-α production. In contrast to phenelzine’s effect, serotonin increased the expression of genes related to ROS and RNS production while reducing IL-6 and TNF-α production. It is important to note that previous studies have reported both anti-inflammatory and pro-inflammatory effects of serotonin on macrophage cells.

Serotonin has been shown to reduce pro-inflammatory cytokine release from macrophages and promote M2 polarization, suggesting an anti-inflammatory role [29]. Similarly, a study on murine RAW264.7 cells also indicates that serotonin and its metabolites decrease oxidative stress and inflammatory cytokine production such as IL-6 and TNF-α [30]. However, serotonin's effects on immune cells are context-dependent, with some studies indicating pro-inflammatory actions through specific receptors and pathways, while others highlight its anti-inflammatory potential. This underscores serotonin's complex role in immune regulation, varying with specific conditions and signaling contexts [20].

Limitations

Our study has several limitations that should be considered. We utilized RAW264.7 cells, which may not fully replicate the complex biological environment of patients with autoimmune, inflammatory, and cancerous diseases or healthy individuals. Consequently, the findings may not be directly translatable to clinical settings. Additional techniques and tests could have been employed to confirm ROS production more robustly. Our study did not measure other potentially relevant factors, such as LSD1 and additional cytokines, which could offer deeper insights into the molecular mechanisms at play. Despite these limitations, our findings provide valuable insights and lay the groundwork for further research. Our study reveals that phenelzine impacts the inflammatory response and gene expression through multiple mechanisms, with serotonin availability shaping its effects.

## Conclusions

Our study demonstrates that mechanisms such as AHR activation and modulation of reactive oxygen and nitrogen species production, along with the increased availability of serotonin due to phenelzine treatment, can significantly impact the inflammatory state. These mechanisms warrant further consideration and investigation. Additional research, particularly focusing on factors such as LSD1 activation and the pro-inflammatory and anti-inflammatory components involved under varying conditions and cell types, can provide valuable insights into these processes and the effects of phenelzine on patients with autoimmune, inflammatory, and cancerous diseases. Our results revealed that phenelzine increased the production of inflammatory cytokines in the absence of serotonin but had no effect on inflammatory cytokines at moderate and high serotonin concentrations.
